# Marine Derived Hamacanthins as Lead for the Development of Novel PDGFRβ Protein Kinase Inhibitors

**DOI:** 10.3390/md11093209

**Published:** 2013-08-26

**Authors:** Boris Pinchuk, Eugen Johannes, Sheraz Gul, Joachim Schlosser, Christoph Schaechtele, Frank Totzke, Christian Peifer

**Affiliations:** 1 Institute of Pharmacy, University of Kiel, Gutenbergstraße 76, Kiel D-24118, Germany; E-Mails: bpinchuk@pharmazie.uni-kiel.de (B.P.); ejohannes@pharmazie.uni-kiel.de (E.J.); jschlosser@pharmazie.uni-kiel.de (J.S.); 2 European ScreeningPort GmbH, Schnackenburgallee 114, Hamburg D-22525, Germany; E-Mail: sheraz.gul@screeningport.com; 3 ProQinase GmbH, Breisacherstraße 117, Freiburg D-79106, Germany; E-Mails: c.schaechtele@proqinase.com (C.S.); f.totzke@proqinase.com (F.T.)

**Keywords:** marine sponge derived hamacanthins, pyrazin-2(1*H*)-ones, receptor tyrosine kinases, PDGFR inhibitors, anti-cancer activity

## Abstract

In this study, we report on pyrazin-2(1*H*)-ones as lead for the development of potent adenosine triphosphate (ATP) competitive protein kinase inhibitors with implications as anti-cancer drugs. Initially, we identified the pyrazin-2(1*H*)-one scaffold from hamacanthins (deep sea marine sponge alkaloids) by Molecular Modeling studies as core binding motif in the ATP pocket of receptor tyrosine kinases (RTK), which are validated drug targets for the treatment of various neoplastic diseases. Structure-based design studies on a human RTK member PDGFR (platelet-derived growth factor receptor) suggested a straight forward lead optimization strategy. Accordingly, we focused on a Medicinal Chemistry project to develop pyrazin-2(1*H*)-ones as optimized PDGFR binders. In order to reveal Structure-Activity-Relationships (SAR), we established a flexible synthetic route via microwave mediated ring closure to asymmetric 3,5-substituted pyrazin-2(1*H*)-ones and produced a set of novel compounds. Herein, we identified highly potent PDGFR binders with IC_50_ values in an enzymatic assay below µM range, and possessing significant activity against PDGFR dependent cancer cells. Thus, marine hamacanthin-derived pyrazin-2(1*H*)-ones showing interesting properties as lead for their further development towards potent PDGFR-inhibitors.

## 1. Introduction

**Figure 1:**
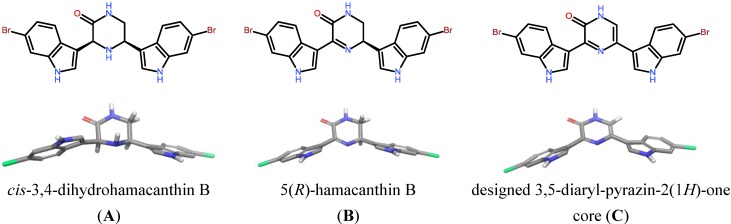
Chemical 2D-representation and energy minimized (Schrödinger Suite 2011: MacroModel, version 9.9, Schrödinger, LLC, New York, NY, 2012) 3D-structures of hamacanthin family members, and 3,5-bis(6-bromo-1*H*-indol-3-yl)-1*H*-pyrazin-2-one as the concept of this study. The compounds formally show different oxidation status of the piperazine-2-one core with significant impact to 3,5-diaryl-conformational parameters.

Marine-derived bioactive compounds and their novel chemical scaffolds have been shown to be attractive starting points for drug discovery programs [1,2,3]. In this regard, we became interested in the marine alkaloid family of hamacanthins [4,5,6]. In the course of our work to develop ATP-competitive receptor tyrosine kinase (RTK) inhibitors with anti-cancer activity [7,8,9,10], we focused on the deep-sea sponge derived bis-indole alkaloids possessing a 3,5-bis-indole-piperazin-2-one and 3,5-bis-indole-3, 4-dihydropyrazin-2(1*H*)-one scaffold, respectively (Figure 1) [4,5,11,12]. Recently, *cis*-3,4-dihydro hamacanthin B was reported to be a potent bacterial methicillin-resistant *Staphylococcus* protein kinase (PK) inhibitor (MRSA-PK inhibitor) with an IC_50_ value of 0.016 µM and significant selectivity over human protein kinase isoforms [13]. Furthermore, the 2(1*H*)-pyrazinone scaffold is present as core moiety in PK inhibitors [14]. Among human PK are validated drug targets in oncology; over-activated RTK including VEGFR, PDGFR and c-kit are considered to be major targets for the development of clinically effective inhibitors [15,16,17]. In line with this notion, many anti-cancer compounds that are advanced into the clinic show (group-) selectivity towards VEGFR, FGFR, EGFR, PDGFRα/β, c-kit, and Flt-3 [18]. Since all PKs from the kinome use ATP as a cofactor for the phosphorylation of proteins in signal transduction pathways, they share a highly conserved ATP binding pocket that is the molecular binding site of most PK inhibitors [19]. Thus, small molecular differences in amino acid identities adjacent to the ATP pocket provide selectivity filters for specific inhibitors [20]. Therefore, we hypothesize that the hamacanthin-core could serve as a suitable scaffold for the design of specific PK inhibitors.

Our molecular modeling and docking approaches for the ATP binding pocket of the RTK VEGF-R2 suggests that only the 3,5-diaryl-pyrazin-2(1*H*)-one core (Figure 1C) is capable of occupying the narrow ATP active site of this RTK. Furthermore, the pyrazin-2(1*H*)-one core was reported to be a key binding motif to PK [14]. In contrast, neither the 3,5-bis-indole-piperazin-2-one nor the 3,5-bis-indole-3,4-dihydropyrazin-2(1*H*)-one core of hamacanthins produced plausible docking poses. The lack of reasonable binding modes in our modeling studies is mainly due to the chiral center(s) of the core scaffold positioning the 3,5-bis-indole-moieties out of the piperazin-2-one ring-plane, and the 5-indole moiety out of the 5,6-dihydropyrazin-2(1*H*)-one ring plane, respectively (Figure 1A,B). Our observation is in line with the notion that *cis*-3,4-dihydrohamacanthin B was reported to be an allosteric MRSA-PK inhibitor that addresses a tetrameric interface region of the PK-protein, and was not determined by X-ray analysis as ligand binding to the ATP site [4].

**Figure 2:**
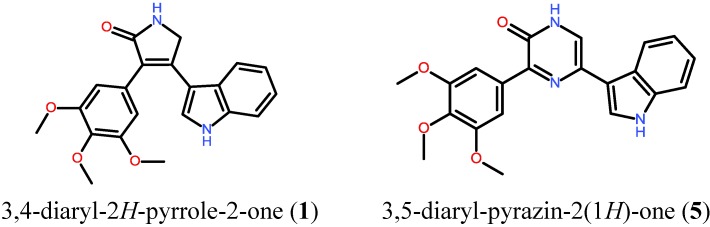
Structures of the reported potent VEGF-R2/3 inhibitor, compound **1** and the newly designed and synthesized compound **5** of this study showing comparable decoration patterns of the aryl moieties.

In this study, we designed the aryl-substitution pattern of the 6-membered 3,5-diaryl-pyrazin-2(1*H*)-one (compound **5**) based on the corresponding 5-membered 3,4-diaryl-2*H*-pyrrole-2-one (compound **1**, Figure 2), a potent inhibitor developed in a former project showing strong activity against the RTK VEGF-R2/3 (IC_50_ = 0.03 µM) and with good efficacy in cellular assays [9].

## 2. Results and Discussion

### 2.1. Synthesis of Designed Compound **5**

**Figure 3:**
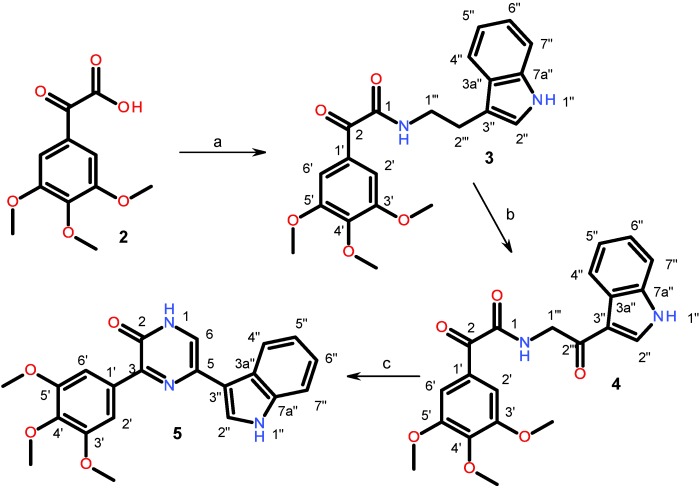
Synthesis of designed compound **5** by microwave mediated ring closure strategy. Reagents and conditions: (**a**) 1. CDI, dichloromethane 2. tryptamine; (**b**) DDQ, THF; (**c**) NH_4_Ac, HAc, microwave heating for 5 min.

In order to confirm the hypothesis that the pyrazin-2(1*H*)-ones are suitable scaffolds for PK inhibitor development, we synthesized compound **5** that involved a modified microwave-mediated ring closure strategy (Figure 3) [12]. In summary, glyoxylic acid **2** was activated by carbonyldiimidazole (CDI) and coupled with tryptamine to produce compound **3**, which upon DDQ-oxidation yielded compound **4**. Ring closure in the final step to produce the targeted pyrazin-2(1*H*)-one compound **5** was straightforward and involved an optimized microwave-mediated reaction using ammonium acetate as the nitrogen source.

### 2.2. Biological Evaluation: Activity against PKs

Interestingly, in a preliminary screen [21] involving 24 therapeutically relevant PK targets, compound **5** inhibited VEGF-R2/3 in the low µM range, and most potently inhibited PDGFRβ with an IC_50_ of 0.5 μM. Moreover, compound **5** was shown to exhibit promising selectivity over the other PK enzymes tested as part of the panel (Table 1). Thus, in order to further enhance potency of this lead compound towards the PDGFRβ drug target [22] we used a structure-based optimization approach. As no X-ray structure of the PK domain of PDGFR was available in the public domain, we generated a homology model of PDGFRβ based on the highly related RTK VEGF-R2 as template structure (pdb code 2p2h [23] using Schrödinger Prime, version 3.1, Schrödinger, LLC, New York, NY, USA, 2012).

**Table 1:** IC_50_ values (µM) of compounds **1** and **5** in a panel [21] of 24 therapeutically relevant PK targets (- indicates no significant PK inhibition was observed at a compound concentration of 100 µM).

Protein kinase	IC_50_ (µM) for Compound 1	IC_50_ (µM) for Compound 5
AKT1	-	-
ARK5	16	-
Aurora-A	46	-
Aurora-B	66	-
B-RAF-VE	-	-
CDK2/CycA	66	-
CDK4/CycD1	56	-
COT	32	-
EGFR	23	-
EPHB4	53	-
ERBB2	51	-
FAK	9	-
IGF1R	22	-
SRC	14	-
VEGF-R2	0.031	4
VEGF-R3	0.037	5
FLT3	61	36
INSR	43	-
MET	60	-
PDGFRβ	11	0.5
PLK1	-	-
SAK	10	-
TIE2	5	-
CK2a1	-	-

### 2.3. Molecular Modeling

**Figure 4:**
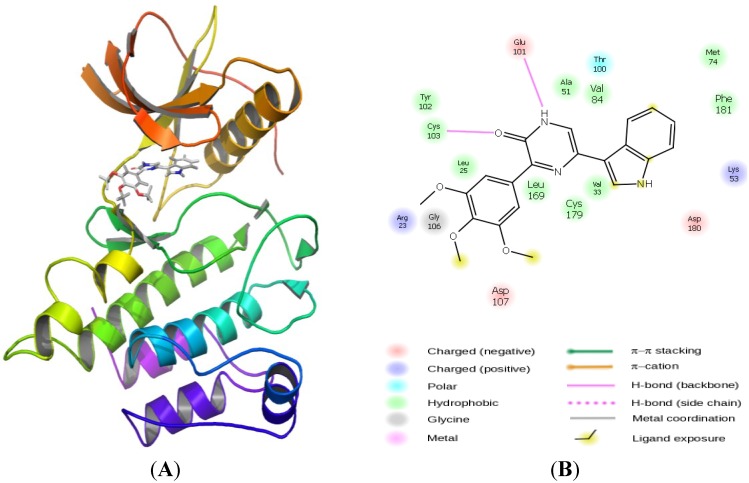
(**A**) Homology model of PDGFRβ based on template structure of VEGF-R2 (pdb code 2p2h [23]) and modeled binding mode of compound **5** in the ATP pocket. (**B**) Ligand-interaction diagram of compound **5** in the ATP binding site of the PDGFRβ homology model. Key amino acid residues and hydrogen bonds are shown (see legend).

Docking compound **5** into the ATP binding site of our homology model of PDGFRβ revealed an interesting and reasonable binding mode (Figure 4, details of the methodology can be found in Section 3.2). The key features of the interaction of compound **5** include the hydrogen bonds formed with the backbone carbonyl of Glu101 and to the amine of Cys103 in the hinge region of the PK, respectively. The indole ring is located in the hydrophobic pocket I whereas the trimethoxyphenyl moiety is located in the solvent exposed hydrophobic region II (in accord with a type-I PK inhibitor [24]).

**Figure 5:**
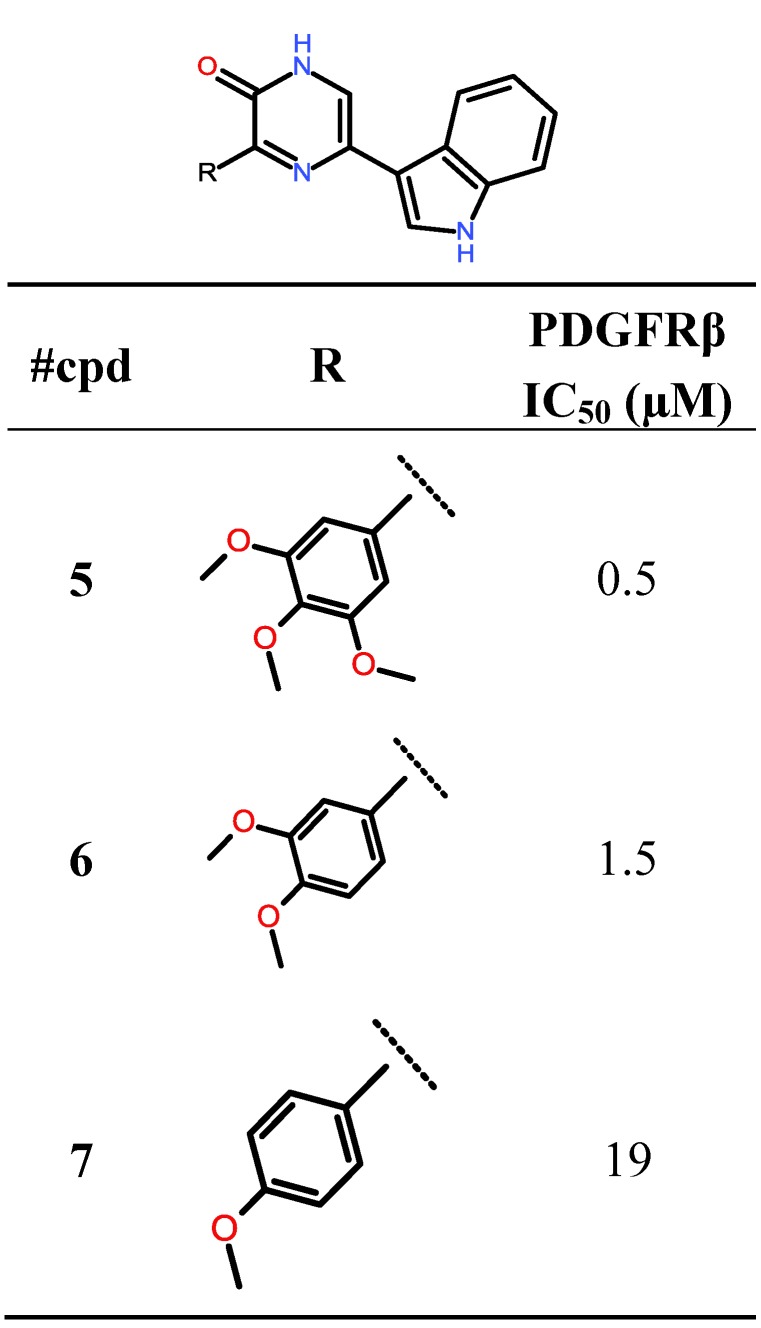
Aryl variation of the pyrazin-2(1*H*)-one 3′-position and PDGFRβ IC_50_ values (µM) of compounds **5**–**7**.

Having demonstrated a rational binding mode for compound **5** in the active site of PDGFRβ, we subsequently aimed to optimize this compound by performing a virtual screen against a focused set of compounds, with particular focus upon varying the indole at the pyrazin-2(1*H*)-one 5′ position, as the pyrazin-2(1*H*)-one 3′-(3,4,5-trimethoxyphenyl) moiety had already been shown to be required in order to inhibit PDGFRβ (Figure 5 [9,25]).

**Figure 6:**
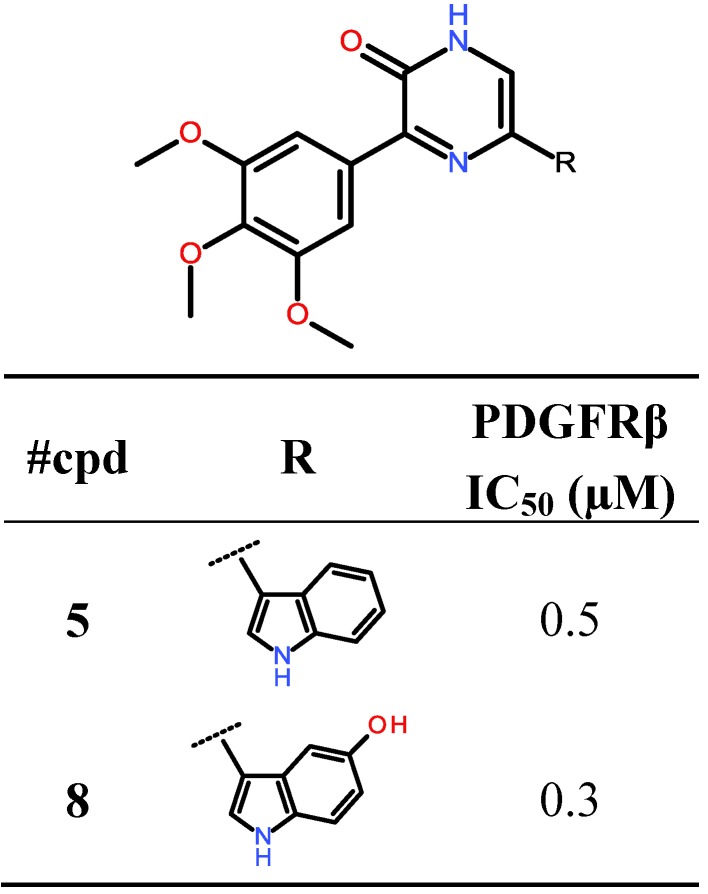
5′OH-Indole variation and PDGFRβ-IC_50_ values (µM) of compounds **5** and **8**.

Thus, the indole moiety originally present in the pyrazin-2(1*H*)-one 5′-position was systematically varied by synthetically feasible decoration patterns yielding a set of virtual compounds. The subsequent ligand preparation, docking and scoring campaign using Schrödinger Glide SP (Suite 2012 Glide version 5.8, Schrödinger, LLC, New York, NY, USA) produced a list with compound **8** as the one that was predicted to inhibit PDGFRβ most potently (Figure 6). The general binding mode of compound **8** is comparable to the binding pose of compound **5** with the pyrazin-2(1*H*)-one core addressing H-bonds to Glu101 and Cys103 (Figure 4), but with the indole-5′OH situated in the hydrophobic pocket II addressing an additional H-bond to the backbone amide-carbonyl oxygen of Val84.

In accordance with our modeling, compound **8** showed slightly enhanced potency against PDGFRβ in the PDGFRβ assay (IC_50_ value = 0.3 µM, Figure 6).

### 2.4. Biological Activity in Cancer Cell Lines of Compounds **5** and **8**

**Figure 7:**
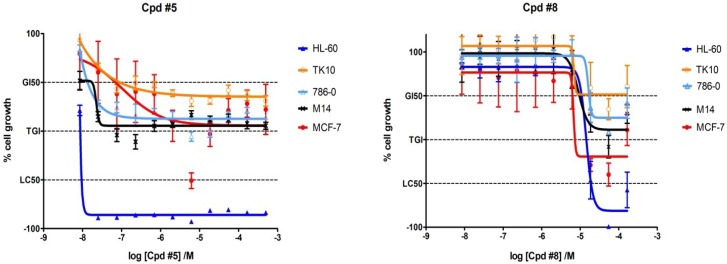
Determination of cytotoxic profiles of compounds **5** and **8** in cell viability assays using HL-60, TK 10, 786-0, M 14, and MCF-7 cell lines.

In order to determine the cytotoxic profiles of compounds **5** and **8**, they were evaluated in cell viability assays (Figure 7) including HL-60, a human myeloblastic leukemia cell line, as it has been shown that the proliferation and differentiation of these cells depend upon PDGFR-signalling [26]. Cells were treated with compounds **5** and **8** and after 48 h incubation, their viability was determined. The compounds exhibited a differentiated cytotoxic profile. Both compounds were shown to be cytotoxic against HL-60 cells such that compound **5** was associated with an IC_50_ value of 0.026 µM and exhibited a significantly stronger anti-proliferative effect than compound **8** that had an IC_50_ value of 30 µM. This is in sharp contrast to the data from isolated PDGFRβ assay and may be due to limited cellular bioavailability of **8**. Interestingly, the other cells tested were significantly less affected. This is in line with the notion that HL-60 cells depend on PDGFR-signaling [26]. However, in our ongoing studies we are currently investigating further molecular details of the reported selectivity of compound **5** against HL-60 cells.

## 3. Experimental Section

### 3.1. Chemistry and Synthesis of Test Compounds

^1^H (300 MHz) and ^13^C (75 MHz) NMR were recorded on a Bruker Avance III 300 spectrometer (Rheinstetten, Germany) at 300 K with a multinuclear probe head using the manufacturer’s pulse programs. The data are reported as follows: chemical shift in ppm from Me_4_Si (TMS) as external standard, multiplicity and coupling constant (Hz). NMR spectra were obtained on a ^1^H (300 MHz) and ^13^C spectra (75 MHz) were referenced either to TMS or to internal DMSO-d_5_ (^1^H NMR δ 2.50) and internal DMSO-d_6_ (^13^C NMR δ 39.5) or internal CHCl_3_ (^1^H NMR δ 7.26) and internal CDCl_3_ (^13^C NMR δ 77.0). All coupling constants (*J* values) are quoted in Hz. The following NMR abbreviations are used: br (broad), s (singlet), d (doublet), t (triplet), m (unresolved multiplet). The labelling scheme of structures to correlate NMR signals can be found in Supporting Information.

Mass spectra of the compounds were recorded after chromatographic separation. Mixtures were separated with an Agilent 1100 HPLC system (Waldbronn; Germany) consisting of a thermostated autosampler, diode array detection and an Agilent Zorbax Eclipse XDB-C8 column (150 × 4.6 mm, 5 µm particle size). Elution was achieved with a solvent gradient system of water and acetonitrile, with 0.1% of acetic acid and a flow rate of 1 mL/min. The eluent flow was splitted to the mass spectrometer.

Mass spectra with nominal resolution were recorded with an Esquire ~LC mass spectrometer (Bruker Daltonik, Bremen, Germany), with electrospray ionization operating in the positive ion mode, with the following parameters: drying gas nitrogen 8 L/min, nebulizer 35 psi, dry gas heating 350 °C, HV capillary 4000 V, HV EndPlate offset −500 V. GC/MS was performed on a HP6890 Series System. EI-Mass spectra were recorded on a Varian MAT 311A (70 eV). HRMS spectra were recorded on a MAT-95 (Finnigan).

Melting points/decomposition temperatures were determined on a Büchi apparatus according to Dr. Tottoli and are uncorrected.

Where appropriate, column chromatography was performed for crude precursors with Merck silica gel 60 (0.063–0.200 mm) or Acros organics silica gel (0.060–0.200 mm; pore diameter *ca.* 60 nm). Column chromatography for test compounds was performed using a La-Flash-System (VWR) with Merck silica gel 60 (0.015–0.040 mm) or RP8 columns. The progress of the reactions was monitored by thin-layer chromatography (TLC) performed with Merck silica gel 60 F-245 plates. Where necessary, reactions were carried out in a nitrogen atmosphere using 4Å molecular sieves. All reagents and solvents were obtained from commercial sources and used as received (THF was used after distillation over K/benzophenone). Reagents were purchased from Sigma-Aldrich Chemie, Steinheim, Germany; Lancaster Synthesis, Mühlheim, Germany or Acros, Nidderau, Germany.

HPLC analysis was performed on a Hewlett-Packard HP 1090 Series II using a Thermo Betasil C8 (150 × 4.6, 5 µM) column (mobile phase flow 1.5 mL/min, gradient KH_2_PO_4_ buffer pH 2.3/methanol, UV-detection 230/254 nm). All key compounds were proven by this method to show ≥98% purity. 

#### 3.1.1. Synthesis of Compound **3**

CDI (1.1 equivalent) was added to a solution of 1 equivalent 2-oxo-2-(3,4,5-trimethoxyphenyl)acetic acid (**2**) in *N*-methylpyrrolidone and reacted at room temperature for 1 h. Then, tryptamine (1 equiv) was added and the mixture was stirred overnight. Then, water was added to quench the reaction and the mixture was extracted three times with EtOAc. The combined organic phase was evaporated under reduced pressure and the residue purified by flash silica gel chromatography to afford *N*-[2-(1H-indol-3-yl)ethyl]-2-oxo-2-(3,4,5-trimethoxyphenyl)acetamide (**3**). Yield: (2.2 g, 91%); mp 119 °C; ^1^H NMR (300 MHz, DMSO-*d*_6_) δ: 2.98 (t, *J* = 7.3 Hz, 2H, CH_2_-2′″), 3.57 (dt, *J* = 7.1, 6.1 Hz, 2H, CH_2_-1′″), 3.77 (s, 9H, 3 OMe), 6,98 (t, *J* = 6.9 Hz, 1H, H-5″), 7.07 (t, *J* = 7.0 Hz, 1H, H-6″), 7.20 (d, *J* = 2.3 Hz, 1H, H-2″), 7.28 (s, 2H, H-2′,6′), 7.34 (d, *J* = 8.0 Hz, 1H, H-7″), 7.57 (d, *J* = 7.7 Hz, 1H, H-4″), 8.99 (t, *J* = 5.75 Hz, 1H, CONH), 10.82 (s, 1H, NH-1″); ^13^C NMR (75 MHz, DMSO-*d*_6_) δ: 24.8 (CH_2_-2′″), 39.1 (CH_2_-1′″), 56.0 (2′,5′-OMe), 60.3 (4′-OMe), 107.3 (CH-2′,6′), 111.3 (CH-7″), 111.4 (C_q_-3″), 118.2 (CH-5″,4″), 120.9 (CH-6″), 122.7 (CH-2″), 127.1 (C_q_-3a″), 128.0 (C_q_-1′), 136.2 (C_q_-7a″), 143.1 (C_q_-4′), 152.8 (C_q_-3′,5′), 164.6 (CO-1), 188.9 (CO-2); LC-MS *m/z* 383 [M + H]^+^.

#### 3.1.2. Synthesis of Compound **4**

To a solution of **3** in THF/H_2_O (9:1) at 0 °C, DDQ (1.5 equiv. dissolved in THF) was added dropwise and stirred for 1 h. Then the solvent was evaporated to dryness. To the residual mixture, methanol was added. The precipitate was filtered off and washed with H_2_O and methanol to afford *N*-[2-(1*H*-indol-3-yl)-2-oxo-ethyl]-2-oxo-2-(3,4,5-trimethoxyphenyl)acetamide (**4**). Yield: (363 mg, 58%); mp 201 °C; ^1^H NMR (300 MHz, DMSO-*d*_6_) δ: 3.80 (s, 3H, OMe), 3.93 (s, 6H, 2 OMe), 4.69 (d, *J* = 6.0 Hz, 2H, CH_2_-1″), 7.23 (m, 2H, H-5″,6″), 7.51 (m, 1H, H-7″), 7.57 (s, 2H, H-2′,6′), 8.16 (m, 1H, H-4″), 8.51 (d, *J* = 3.15 Hz, 1H, H-2″), 9.21 (t, *J* = 5.9 Hz, 1H, CONH), 12.08 (s, 1H, NH-1″); ^13^C NMR (75 MHz, DMSO-*d*_6_) δ: 45.4 (CH_2_-1′″), 56.1 (2′,5′-OMe), 60.3(4′-OMe), 107.5 (CH-2′,6′), 112.2 (CH-7″), 113.8 (C_q_-3″), 121.0 (CH-4″), 122.0, 122.9 (CH-5″,6″), 125.4 (C_q_-3a″), 128.2 (C_q_-1′), 133.9 (CH-2″), 136.4 (C_q_-7a″), 143.1 (C_q_-4′), 152.9 (C_q_-3′,5′), 166.3 (CO-1), 189.1 (CO-2), 189.7 (CO-2′″); LC-MS *m/z* 397 [M + H]^+^.

General procedure for pyrazinone ring closure using microwave synthesis (compounds **5**, **6** and **8a**) [27].

A microwave vial (5 mL) was equipped with ammonium acetate (10 equiv) and a solution of diketone **4** [27] (1 equiv) in acetic acid (3 mL). The vial was sealed and stirred at 160 °C for 4 min in a microwave synthesizer (CEM Discover). The reaction vessel was cooled to rt when H_2_O was added to precipitate the pyrazinone, which was filtered off. The pyrazinone was purified by preparative HPLC (RP-phase) to afford the test compound ≥98% purity.

#### 3.1.3. Synthesis of Compound **5**

By using the general procedure for pyrazinone ring closure we obtained 5-(1*H*-indole-3-yl)-3-(3,4,5-trimethoxyphenyl)pyrazin-2(1*H*)-one (**5**). Yield after final purification: (31 mg, 11%); mp 293 °C; ^1^H NMR (300 MHz, DMSO-*d*_6_) δ: 3.76 (s, 3H, OMe), 3.88 (s, 6H, 2 OMe), 7.12 (m, 2H, H-5″,6″), 7.44 (m, 1H, H-7″), 7.90 (s, 1H, H-6), 7.92 (d, *J* = 2.6 Hz, 1H, H-2″), 8.01 (s, 2H, H-2′,6′), 8.30 (d, *J* = 7.4 Hz, 1H, H-4″), 11.34 (s, 1H, NH-1″), 12.53 (s, 1H, NH-1); ^13^C NMR (75 MHz, DMSO-*d*_6_) δ: 55.8 (2′,5′-OMe), 60.1 (4′-OMe), 106.0(CH-2′,6′), 111.8 (C_q_-7″) 112.1(C_q_-3″), 119.4, 120.4, 121.5 (CH-4″,5″,6″), 123.1 (CH-2″,6), 124.8 (C_q_-3a″), 131.9 (C_q_-1′,7a″), 136.7 (C_q_-3,5), 138.9 (C_q_-4′), 152.3 (C_q_-3′,5′), 154.1 (CO-2); LC-MS *m/z* 378 [M + H]^+^. HRMS: *m/z* calculated for [M]^+^ C_21_H_19_N_3_O_4_: 377.1375; found 377.1363.

#### 3.1.4. Synthesis of Compound **6**

By using general procedure for pyrazinone ring closure, 3-(3,4-dimethoxyphenyl)-5-(1*H*-indol-3-yl)-1*H*-pyrazin-2-one (**6**) was synthesized from *N*-[2-(1H-indole-3-yl)-2-oxoethyl]-2-(3,4-dimethoxyphenyl)-2-oxoacetamide [27]. Yield: (50 mg, 51%); mp 259 °C; ^1^H NMR (300 MHz, DMSO-*d*_6_) δ: 3.84 (s, 3H, OMe), 3.87 (s, 3H, OMe), 7.12 (m, 3H, H-3′,5″,6″), 7.44 (d, *J* = 7.4 Hz, 1H, H-7″), 7.81 (s, 1H, H-6), 7.90 (d, *J* = 2.6 Hz, 1H, H-2″), 8.18 (d, *J=* 1.9 Hz, 1H, H-6′), 8.25 (d, *J=* 7.7 Hz, 1H, H-4″), 8.30 (dd, *J=* 8.6, 1.9 Hz, 1H, H-2′), 11.34 (s, 1H, NH-1″), 12.40 (s b, 1H, NH-1); ^13^C NMR (75 MHz, DMSO-*d*_6_) δ: 55.3, 55.5 (2 OMe), 110.9 (CH-3′), 111.4 (CH-6″), 111.8 (CH-7″), 112.3 (C_q_-3″), 119.4 (CH-6′), 120.4 (CH-4″), 121.4 (CH-5″), 122.1 (CH-2′), 123.1 (CH-2″,6), 124.8 (C_q_-3a″), 129.3 (C_q_-1′), 136.7 (C_q_-7a″), 148.1 (C_q_-3″,5″,5′), 150.2 (C_q_-4′), 154.1 (CO-2); LC-MS *m/z* 348 [M + H]^+^. HRMS: *m/z* calculated for [M]^+^ C_20_H_17_N_3_O_3_: 347.1270; found: 347.1254.

#### 3.1.5. Synthesis of Compound **7**

By using general procedure for pyrazinone ring closure, 3-(4-methoxyphenyl)-5-(1*H*-indol-3-yl)-1*H*-pyrazin-2-one (**7**) was synthesized from *N*-[2-(1H-indole-3-yl)-2-oxoethyl]-2-(4-methoxyphenyl)-2-oxoacetamide [27]. Yield: (200 mg, 58%); mp 232 °C; ^1^H NMR (300 MHz, DMSO-*d*_6_) δ: 3.84 (s, 3H, OMe), 7.06 (d, *J* = 9.1 Hz, 2H, H-3′,5′), 7.10-7.18 (m, 2H, H-5″,6″), 7.43 (d, *J* = 7.1 Hz, 1H, H-7″), 7.78 (s, 1H, H-6), 7.88 (d, *J* = 2.6 Hz, 1H, H-2″), 8.15 (d, *J* = 7.2 Hz, 1H, H-4″), 8.50 (d, *J* = 9.0 Hz, 2H, H-2′,6′), 11.34 (s, 1H, NH-1″), 12.42 (s, 1H, NH-2); ^13^C NMR (75 MHz, DMSO-*d*_6_) δ: 55.2 (OMe), 111.7 (CH-7″), 112.3 (C_q_-3″), 113.4 (CH-3′,5′), 119.5 (CH-6″), 120.2 (CH-7″), 121.4 (CH-5″), 123.2 (CH-2″,6), 124.7 (C_q_-3a″,5), 129.1 (C_q_-1′), 130.0 (CH-2′,6′), 136.7 (C_q_-3,7a″), 154.2 (CO-2), 160.3 (C_q_-4′); LC-MS *m/z* 318 [M + H]^+^. HRMS: *m/z* calculated for [M]^+^ C_19_H_15_N_3_O_2_: 317.1164; found: 317.1175.

#### 3.1.6. Synthesis of Compound **8a**

By using general procedure for pyrazinone ring closure 5-(5-benzyloxy-1*H*-indol-3-yl)-3-(3,4,5-trimethoxyphenyl)-1*H*-pyrazin-2-one, compound **8a** was synthesized from *N*-[2-(5-benzoxy-1H-indol-3-yl)-2-oxo-ethyl]-2-oxo-2-(3,4,5-trimethoxyphenyl)acetamide [27]. Yield: (220 mg, 76%); mp 231 °C; ^1^H NMR (300 MHz, DMSO-*d*_6_) δ: 3.73 (s, 3H, OMe), 3.81 (s, 6H, 2 OMe), 5.09 (s, 2H, CH_2_), 6.93 (dd, *J* = 8.8, 2.40 Hz, 1H, H-6″), 7.32–7.46 (m, 6H, H-7″, 5 H-Bn), 7.88 (s, 1H, H-6), 7.90 (d, *J* = 2.5 Hz, 2H, H-2″,4″), 7.97 (s, 2H, H-2′,6′), 11.22 (d, *J* = 2.5 Hz, 1H, NH-1″), 12.36 (s, 1H, NH-1); ^13^C NMR (75 MHz, DMSO-*d*_6_) δ: 55.9 (2 OMe), 60.1 (OMe), 70.3 (CH_2_), 105.4 (CH-4″), 106.2 (CH-2′,6′), 111.1 (CH-6″), 111.7 (C_q_-3″), 112.1 (CH-7″), 124.0 (CH-2″,6), 125.3 (C_q_-3a″), 127.7, 127.7, 128.3 (5 CH-Bn), 132.0 (C_q_-7a″), 132.2 (C_q_-1′,3a″), 137.6 (C_q_-3,5), 139.0 (C_q_-4′), 152.3 (C_q_-3′,5′), 152.8 (C_q_-5″), 154.1 (CO-2); LC-MS *m/z* 484 [M + H]^+^. HRMS: *m/z* calculated for [M]^+^ C_28_H_25_N_3_O_5_: 483.1794; found: 483.1776.

#### 3.1.7. Synthesis of Compound **8**

A microwave vial (5 mL) was equipped with compound **8a** (100 mg), cyclohexene (90 mg), Pd/C 10% (20 mg) and methanol (1.5 mL). The vial was sealed and stirred 10 min at 100 °C in a microwave synthesizer. The solution was filtered and cooled to room temperature to form a precipitate which was filtered and washed with methanol and Et_2_O to afford 5-(5-hydroxy-1*H*-indole-3-yl)-3-(3,4,5-trimethoxyphenyl)pyrazin-2(1*H*)-one, compound **8**. Yield: (52 mg, 64%); mp 340 °C; ^1^H NMR (300 MHz, DMSO-*d*_6_) δ: 3.76 (s, 3H, OMe), 3.88 (s, 6H, 2 OMe), 6.68 (dd, *J* = 8.7, 2.33 Hz, 1H, H-6″), 7.23 (d, *J* = 8.6 Hz, 1H, H-7″), 7.64 (d, *J* = 2.2 Hz, 1H, H-4″), 7.79 (s, 1H, H-6), 7.80 (d, *J* = 2.7 Hz, 1H, H-2″), 8.00 (s, 2H, H-2′,6′), 8.67 (s, 1H, OH), 11.05 (d, *J* = 2.4 Hz, 1H, NH-1″), 12.47 (s, 1H, NH-1); ^13^C NMR (75 MHz, DMSO-*d*_6_) δ: 55.7 (2 OMe), 60.1 (OMe), 104.7 (CH-4″), 106.0 (CH-2′,6′), 111.3 (C_q_-3″), 111.8, 111.9 (CH-6″,7″), 123.5 (CH-2″,6), 125.7 (C_q_-4′), 131.9 (C_q_-1″,7a″), 132.8 (C_q_-3a″), 138.8 (C_q_-4′), 151.2 (COH-5″), 152.3 (C_q_-3′,5′), 154.0 (CO-2); LC-MS *m/z* 394 [M + H]^+^. 

### 3.2. Molecular Modeling

All modeling was performed on a DELL 8 core system. For visualization and building the structures Maestro (version 9.3) from Schrödinger (Schrödinger, LLC, New York, NY, USA, 2012) was used (VEGF-R2 pdb code 2p2h, [23]). The illustrations of modeling were generated by Maestro. For compound docking and screening the Schrödinger “Glide SP” workflow was used [28]. The goal of the Glide methodology is to semiquantitatively rank the ability of candidate ligands to bind to a specified conformation of the protein receptor. Prior to determining binding poses of ligands energetically minimized compound conformations were generated, docked into the active site and subsequently ranked based on their calculated binding affinity. 

### 3.3. Biological Evaluation

All inhibitor solutions were prepared freshly in DMSO prior to each experiment and used immediately.

#### 3.3.1. Selectivity Profiling of Compounds by IC_50_ Values Using 24 Protein Kinases

Recombinant protein kinases. The inhibitory profile of compounds was determined using the following 24 protein kinases (GenBankAcc.No. available on http://www.proqinase.com/pages/science [29]): AKT1, ARK5, Aurora-A, Aurora-B, B-Raf-VE, CDK2/CycA, CDK4/CycD1, CK2-A1, EGF-R, EPHB4, ERBB2, FAK, IGF1-R, SRC, VEGF-R2, VEGF-R3, FLT3, INS-R, MET, PDGFRβ, PLK1, SAK, TIE2 and COT. All protein kinases were expressed using human cDNAs in Sf9 insect cells as recombinat GST-fusion proteins or His-tagged proteins by means of the baculovirus expression system. Kinases were purified by affinity chromatography using either GSH-agarose (Sigma) or Ni-NTA-agarose (Qiagen). The purity and identity of each kinase was determined by SDS-PAGE/silver staining and western blot analysis using specific antibodies.

Protein kinase Assay. A proprietary protein kinase assay (^33^PanQinase^®^ Activity Assay) was used for measuring the kinase activity of the 24 protein kinases. All protein kinase assays were performed in 96-well FlashPlates™ (Perkin Elmer/NEN, Boston, MA, USA) in a 50 µL reaction volumes. Assays for all enzymes were performed in a solution containing 60 mM HEPES-NaOH, pH 7.5, 3 mM MgCl_2_, 3 mM MnCl_2_, 3 µM Na-orthovanadate, 1.2 mM DTT, 50 µg/mL PEG20000, 1 µM [γ-33P]-ATP (approx. 5 × 105 cpm per well), recombinant protein kinase (50–400 ng). Depending upon the kinase being assayed, appropriate substrates were used and were as follows (substrates shown in parentheses): AKT1 (GSK3/14-27), ARK5 (autophosphorylation), Aurora-A, Aurora-B (Tetra(LRRWSLG)), B-Raf-VE (MEK1 KM), CDK2/CycA (histone H1), CDK4/CycD1 (Rb-CTF), CK2-A1 (Casein), EGF-R, EPHB4, ERBB2, FAK, IGF1-R, SRC, VEGF-R2, VEGF-R3 (poly(Glu,Tyr) 4:1), FLT3, INS-R, MET, PDGFRβ (poly(Ala,Glu,Lys,Tyr) 6:2:5:1), PLK1 (Casein), SAK (autophosphorylation), TIE2 (poly(Glu,Tyr) 4:1), COT (autophosphorylation). The IC_50_ values were measured by testing 10 concentrations of compounds by single sampling. The final DMSO concentration in the assay was 1% (v/v). The data were fitted using the 4-parameter logistic fit option of GraphPad Prism 5.

#### 3.3.2. Cell Culture and Proliferative Assays using HL-60, TK 10, 786-0, M 14, and MCF-7 Cells

The cells were grown in RPMI 1640 Glutamax with 10% FCS, 100 µg/mL streptomycin and 100 U/mL Penicillin G and incubated in a 5% CO_2_ humidified atmosphere at 37 °C. For proliferation experiments, cells were seeded in 20 µL pro well into 384-well Greiner 384 CellStar^®^ plates (Greiner Bio-One I. AG, Kremsmünster, AT). In addition to the test plates, one plate was prepared for the reference measurement at day zero. All plates were incubated for 24 h at 37 °C in a humidified atmosphere with 5% CO_2_. Compounds **5** and **8** that were dissolved in 100% DMSO (v/v) were added to test plates using the Echo 550^®^ Liquid Handler (Labcyte Inc., Sunnyvale, UK). The final DMSO concentration in the assay was 0.5% v/v. The viability of the cells in the day zero control plates were determined on the same day without adding any compounds. The CellTiter-Glo^®^ Viability Assay was used to determine the viability of cells using the standard protocol for this assay (Promega Corp., Madison, WI, USA). The luminescence signal was measured at the EnSpire^®^ Multimode Plate Reader (PerkinElmer, Waltham, MA, USA). Test plates were incubated for further 48 h and the cell viability was defined as just described. Measured raw data were converted into percent of cell growth by using the high control (0.5% DMSO v/v without compound) and the day zero control. For dose-response studies, 11 different concentrations of compounds were tested in quadruplicates. The IC_50_ values were calculated using the 4-parameter logistic fit option of GraphPad Prism 5.

## 4. Conclusions

In this study, we developed pyrazin-2(1*H*)-ones as potent and PDGFRβ inhibitors based on marine derived hamacanthins. Modeling studies showed the core moiety of hamacanthins to bind in the ATP binding pocket of RTK and suggested a straightforward strategy towards potent PDGFRβ binders. For subsequent optimization of hamacanthin derivatives as PDGFRβ inhibitors, a flexible synthetic route via microwave-mediated ring closure to asymmetric 3,5-substituted pyrazin-2(1*H*)-ones was established and a set of novel compounds was produced. Herein, we identified highly potent PDGFRβ binders with IC_50_ values in an enzymatic assay below µM range with compound **5** possessing significant activity against PDGFR dependent cancer cells. Thus, marine hamacanthin-derived pyrazin-2(1*H*)-ones showed interesting properties as lead for the further development of highly potent and selective PDGFRβ-inhibitors.
